# Multiple Pleomorphic Tetramers of Thermostable Direct Hemolysin from *Grimontia hollisae* in Exerting Hemolysis and Membrane Binding

**DOI:** 10.1038/s41598-019-46354-x

**Published:** 2019-07-08

**Authors:** Yu-Kuo Wang, Sheng-Cih Huang, Chin-Yuan Chang, Wan-Ting Huang, Man-Jun Liao, Bak-Sau Yip, Feng-Pai Chou, Thomas Tien-Hsiung Li, Tung-Kung Wu

**Affiliations:** 1 Department of Biological Science and Technology, National Chiao Tung University, Hsin-Chu, 30010 Taiwan Republic of China; 2 Center for Emergent Functional Matter Science, National Chiao Tung University, 1001 Ta-Hsueh Rd., Hsinchu, 30010 Taiwan Republic of China; 3 Department of Neurology, National Taiwan University Hospital, Hsin-Chu, 30059 Taiwan Republic of China; 4 Graduate Institute of Biochemistry, National Chung Hsing University, Taichung, 40227 Taiwan Republic of China

**Keywords:** Bacterial toxins, Nanocrystallography

## Abstract

Oligomerization of protein into specific quaternary structures plays important biological functions, including regulation of gene expression, enzymes activity, and cell–cell interactions. Here, we report the determination of two crystal structures of the *Grimontia hollisae* (formally described as *Vibrio hollisae*) thermostable direct hemolysin (Gh–TDH), a pore-forming toxin. The toxin crystalized in the same space group of *P*2_1_2_1_2, but with two different crystal packing patterns, each revealing three consistent tetrameric oligomerization forms called Oligomer–I, –II, and –III. A central pore with comparable depth of ~50 Å but differing in shape and size was observed in all determined toxin tetrameric oligomers. A common motif of a toxin dimer was found in all determined structures, suggesting a plausible minimum functional unit within the tetrameric structure in cell membrane binding and possible hemolytic activity. Our results show that bacterial toxins may form a single or highly symmetric oligomerization state when exerting their biological functions. The dynamic nature of multiple symmetric oligomers formed upon release of the toxin may open a niche for bacteria survival in harsh living environments.

## Introduction

Pore–forming toxins (PFTs), a class of virulence factors widely distributed in both prokaryotic and eukaryotic organisms, are common and effective biological weapons used to attack host cells through disruption of cellular membranes^1–4^. Specifically, bacterial PFTs have received far more attention due to their disease–causing abilities. In all clinical strains of *Grimontia hollisae* (formally described as *Vibrio hollisae*), a thermostable direct hemolysin (Gh–TDH) was identified to be a major virulence factor which causes symptoms such as hypovolemic shock, bacteremia, and septicemia when ingesting contaminated raw seafood or upon wound infection in humans^5–8^. The Gh–TDH shows both genetic and biological similarity to the TDH of *Vibrio parahaemolyticus* (Vp–TDH)^9^. Extensive biochemical and biophysical studies, mostly on Vp–TDH, shows that pore–forming TDH causes colloidal osmosis lysis of erythrocytes^10,11^ and induces cyto–, cardio–, entero–, and hepato–toxicity in various types of cultured cells as well as mouse lethality^12–15^. Unlike the related Vibrio species of *Vibrio cholera* where the identified cell surface receptor is ganglioside G_M1_^16,17^, conclusions related to the specific cell surface receptor for TDH was not uniform and complicated by the types of activities and the cell types studied. Pre–incubating Vp–TDH with G_T1_ or G_D1a_ ganglioside can neutralize mouse–lethal Vp–TDH activity^18^. However, Douet *et al*. suggested in previous work that asialo–G_M2_ ganglioside, in addition to asialo–G_M1_ and lactocerebroside, is also a preferred receptor^19^. The detailed mechanisms of the interplay between cell membrane receptor(s) and TDH induced activities also require further studies as Vp–TDH can cause the hemolysis of erythrocytes in human, rabbit, and mouse, but only weakly in sheep and horse^10,11^; nevertheless, the toxin binding capacities of Vp–TDH on the membranes of these erythrocytes are similar^20^. It is likely that the induced cell lytic, cyto–, entero–, and hepato–activities are not solely dependent on TDH after the pore–forming TDH binds to either raft or non–raft regions of the cell membrane receptor(s) of ganglioside type carbohydrate moieties^1,15,21^.

The tetrameric conformational structure properties of Vp–TDH in solution have been well characterized^22^. The Vp–TDH crystal structure shows a tetramer that with a central pore, which has with a diameter and depth of 23 Å and ~50 Å, respectively^23^. In parallel, Fukui *et al*. studied the “Arrhenius effect” of TDH, characterized by paradoxical responses to heat detoxification at ~60–70 °C and reactivation by additional heating above 80 °C^23^. This observation reveals the reversible fibrillar aggregation nature of TDH similar to amyloid fibrils formation, which implies the possible involvement of multiple interfaces of the TDH protomer in oligomerization^24^. We report herein the crystal structure of Gh–TDH in three tetrameric oligomerization conformations, labeled as Gh-TDH Oligomer–I, –II and –III, respectively. These three Gh–TDH tetrameric oligomers consistently present in two different crystal packing patterns of a same crystallographic space group–*P*2_1_2_1_2, suggesting possible thermodynamic equilibrium in solution instead of the crystal packing effect^25^. The Gh–TDH Oligomer–I structure was indistinguishable from the reconstituted Vp–TDH tetramer structure; whereas Oligomer–II and –III structures gave further insight into multiple Gh–TDH tetrameric oligomerization conformations in erythrocytes binding; the potential interfaces, in addition to those originally found in Oligomer–I structure, in Gh–TDH protomer for protein–protein interaction; and above all, the common TDH dimeric motif found within these Gh–TDH tetrameric pore–forming architectures as a minimum unit in cell membrane binding and probably hemolytic activity.

## Materials and Methods

### Gh–TDH protein purification, crystallization, and structure determination

The purification and crystallization of Gh–TDH protocol has been published elsewhere^11^. Diamond–shaped plate crystals appeared and grew to optimum size of 0.3 × 0.2 × 0.1 mm^3^ in six months. The native and selenomethionyl–derivatized Gh–TDH crystals were flash cooled in a 100 K cold nitrogen–gas stream and their X–ray diffraction data were collected at Biological Crystallography Facility beamline BL13B1 and BL13C1, which are equipped with ADSC Quantum–315r CCD detector, at the National Synchrotron Radiation Research Center (NSRRC, Taiwan)^11^. A number of 30 thin plate crystals were screened for the best quality data with high resolution and low mosaicity (<0.6) along the process. The diffraction images were indexed, integrated, and scaled using HKL2000 program suite (Supplemental Table 1)^26^. Two selenomethionyl–derivatized Gh–TDH data, designated as Gh–TDH–I oligomer and Gh–TDH–II oligomer respectively, which yielded almost identical cell parameters and the same space group *P*2_1_2_1_2 but with different tetramer packing pattern per asymmetric unit, were used in multi–wavelength anomalous dispersion methodology (MAD) for phasing in structure determination^27,28^. The R_work_ for Gh–TDH–I and Gh–TDH–II was 0.2903 and 0.2561 respectively. An additional step of individual anisotropic temperature factor refinement was used only for 1.7 Å resolution of Gh–TDH–I structure before adding water molecules. Solvent molecules were initially added by PHENIX.REFINE using the default settings and then manually checked for appropriate hydrogen–bonding stereochemistry (bond length within 2.5 and 3.5 Å and bond angle greater than 100º) and with electron–density greater than 2.0. A total of 4,817 non–hydrogen atoms and 240 solvent molecules were included in the refined Gh–TDH–I oligomer model with the final R_work_ and R_free_ of 0.201 and 0.234, respectively; and 4,854 non–hydrogen atoms and 184 solvent molecules were included in the refined Gh–TDH–II oligomer model with the final R_work_ and R_free_ of 0.193 and 0.241, respectively. The atomic coordinates and structure factors of two Gh–TDH structures (PDB codes: 4WX3 and 4WX5) have been deposited in the Protein Data Bank, Research Collaboratory for Structural Bioinformatics, Rutgers University, New Brunswick, NJ (http://www.rcsb.org).

### Molecular mass determination of Gh–DH^WT^ and various Gh–TDH^mut^ proteins

Sodium dodecyl sulfate polyacrylamide gel electrophoresis (SDS–PAGE) experiments were performed to determine the purity of the protein and the probable denatured molecular weight^29^. Samples (20 μL) were denatured by 5 X SDS PAGE sample buffer (5 μL containing 2% SDS, 10% glycerol, 125 mM Tris–HCl, pH 6.8, 5% β–mercaptoethanol, and 0.05% bromophenol blue), boiled at 95 °C for 5 min, and subjected to a 15% SDS Polyacrylamide–gel^29^. After electrophoresis performed in fixed 120 voltages, the Coomassie brilliant blue R250 staining solution was used to stain the gel for 20 min. The gel was put in Destaining buffer I (methanol/acetic acid/water = 4:1:5, v/v/v) for 30 min and then Destaining buffer II (methanol/acetic acid/water = 1.2:0.05:8.75) until the protein band was obviously visualized^29^.

The native PAGE was performed on 10% polyacrylamide gel and the procedures were similar to that of SDS PAGE except that the sample buffer and the gels do not contain any SDS and mercaptoethanol and the running condition should be maintained at 4 °C to avoid the protein denaturing^30^. For each well, 20 μL sample was mixed with 4 μL of 5X Native PAGE sample buffer (4 μL containing 10% glycerol, 5% β–mercaptoethanol, and 0.05% bromophenol blue, 125 mM Tris–HCl, pH 6.8) and subjected to electrophoresis immediately with iced 1X Native PAGE running buffer (90 mM Tris–base, 2.5 mM EDTA, 80 mM Boric Acid, pH 8.0) at 90 Volt for 3 h with 4 °C circulating water bath. The proteins were stained and destained in the same steps as SDS–PAGE analysis^30^.

Analytical ultracentrifugation (AUC) analysis of the samples was carried out using a Beckman Coulter ProteomeLab™ XL–A/I analytical ultracentrifuge^31–34^. Briefly, samples were dialyzed against the 20 mM Tris–HCl pH 7.2 buffer prior to analysis. Five–hundreds μL (1 mg/mL) of the Gh–TDH^WT^ or Gh–TDH^mut^ proteins equilibrated in 20 mM Tris–HCl pH 7.2 buffer were subjected to AUC to obtain an apparent distribution of sedimentation coefficients, c(s), and the determined apparent molecular mass (Da). In addition, 500 μL of 20 mM Tris–HCl pH 7.2 buffer alone was used as the reference control. The sample holders were placed in an 8–hole rotor and measurements were run at a constant rotation speed of 42,000 rpm at 20 °C^31–34^. All data were collected at 280 nm.

### Site–directed mutagenesis of *G. hollisae tdh* gene

Site–directed mutagenesis was carried out in plasmid pCR2.1–TOPO–*Gh*–*tdh* using QuikChange site–directed mutagenesis kit (Stratagene). A total of 18 single– or double–mutants including Y53D, F159D, Y53D/F159D, R46E, W39A, W65A, Y87A, F91A, F95A, Y106A, Y107A, F113A, Y87A/Y107A, D78A, K97A, S98A, N108A, and Y126A were prepared. The mutated genes of all Gh–TDH mutants were confirmed by DNA sequencing using ABI PRISM 3730XL auto sequencer (Applied Biosystems) based on dideoxynucleotide chain–termination method. Subsequently, these mutated plasmids were individually transformed into *E. coli* BL21(DE3)pLysS strain, expressed, and purified as Gh–TDH^mut^.

### Hemolytic activity assay

The hemolytic activity was assayed as previously described^11^. Briefly, the rabbit erythrocytes were washed three times with 100 mM PBS buffer pH 7.6 and re–suspended in the same buffer to 4% concentration [vol/vol]^11^. An aliquot of purified Gh–TDH was added and solution was incubated at 37 °C for 60 min. The solution was clarified by centrifugation (500 rpm, 2 min) and the supernatant was measured at 540 nm to determine the resulting hemoglobin concentration. A full scale of the A_540_ absorbance of rabbit erythrocytes mixed with 0.1% Triton X–100 was also determined and defined as 100% hemolysis.

### Cells binding assay of Gh–TDH wild–type and mutants

All tested fluorescent FITC conjugated Gh–TDHs as well as the negative control FTIC–BSA probe were prepared by using the FluoReporter FITC Protein Labeling Kit (Molecular Probes)^15^. HeLa cells were grown in DMEM medium (Invitrogen Inc.) supplemented with 10% fetal bovine serum and 0.1% penicillin/streptomycin. The cells were maintained in a humidified atmosphere consisting of 5% CO_2_ and incubated at 37 °C until use. The HeLa cells were treated with FITC–conjugated Gh–TDH^WT^, Gh–TDH^mut^, or BSA (final concentration 10 μg/ml) for 5 min at 37 °C before washed three times with PBS buffer. The fluorescence of the FITC–conjugated Gh–TDH^WT^, Gh–TDH^mut^, or BSA on Hela cells were monitored by a fluorescence filter cube (excitation/emission at 494/521 nm) on a Nikon TE2000–U Microscope and the image data were recorded on a charge–coupled digital camera.

## Results

### The crystal structures of Gh–TDH tetrameric oligomer

**Figure 1:**
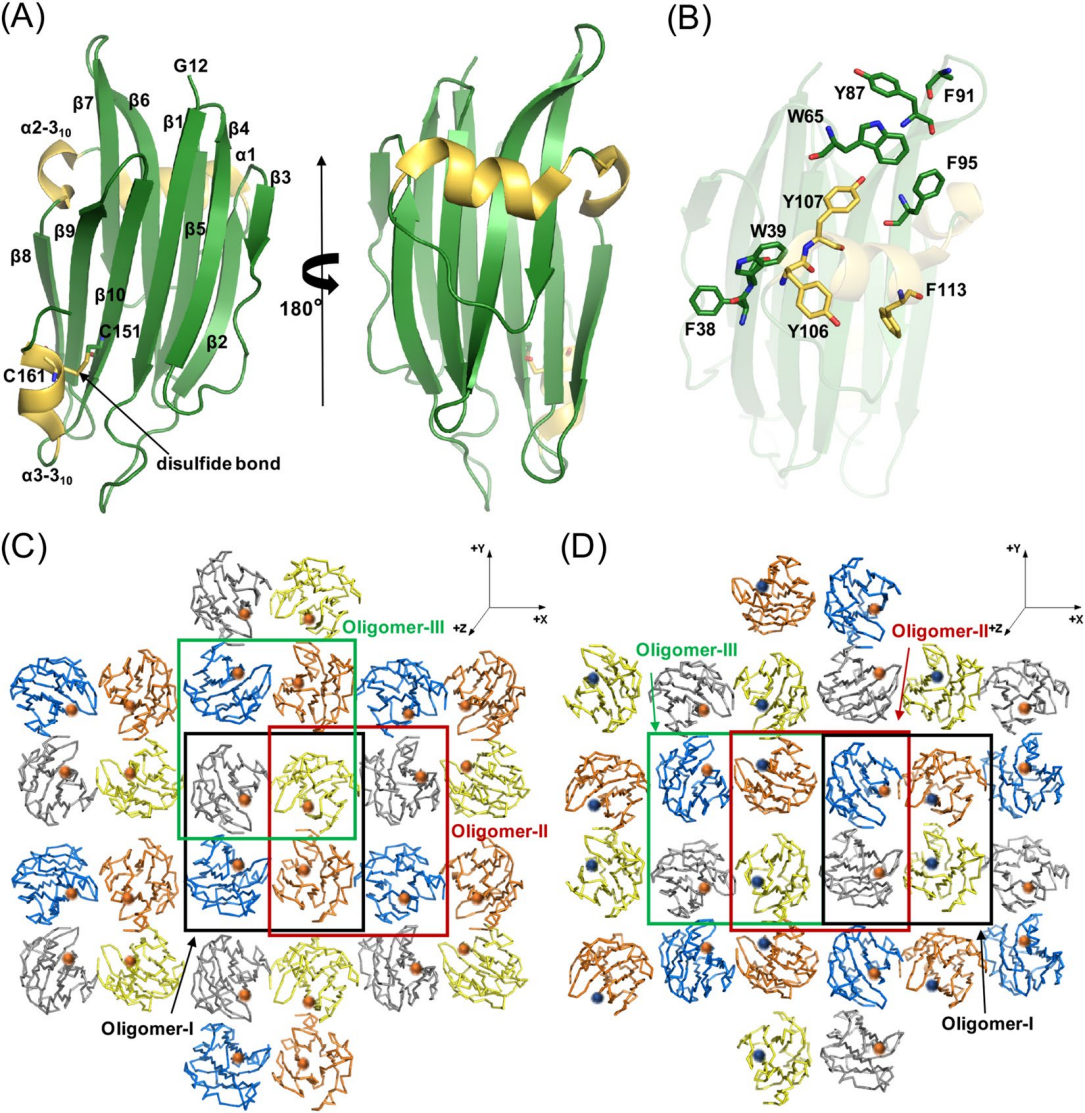
Topological Structure and Crystal Packing Patterns of Gh–TDH Protein. (**A**) Ribbon illustration of the overall structure of Gh–TDH (12–165 a.a.) protomer. (**B**) Residues involved in membrane interaction. (**C**) The crystal packing pattern corresponds to the Gh–TDH–I Oligomer. (**D**) The crystal packing pattern corresponds to the Gh–TDH–II oligomer. The balls in coral or blue color indicate the position and the original orientation of *N*-terminal Gly^12^ of each Gh–TDH protomer. Oligomer–I, –II, and –III were marked within the black, red, and green boxes, respectively.

The crystal structure of the Gh–TDH protomer determined separately at the 1.7 Å and 2.3 Å resolution limit closely resemble the single Vp–TDH protomer structure (PDB entry: 3A57) with a rms deviation 0.4 Å at 154 C_α_ atoms. Both Gh–TDHs adopted the same β–sandwich fold composed of two layers of packed β–pleated sheet of six (strands β1, β3, β4, β8, β9, and β10) and four β strands (strands β2, β5, β6, and β7), flanked by α1 and α2–3_10_ helices (Fig. 1A). An intramolecular disulfide bond between β10 Cys^151^ and 3_10_ helix Cys^161^, which is similar to that of Vp–TDH, was also observed^23^. The full length Gh–TDH composed of 165 amino acid residues in which the first 11 residues (FELPSIPFPSP–) were not observed in the electron density map and presumably retained multiple conformations.

**Figure 2:**
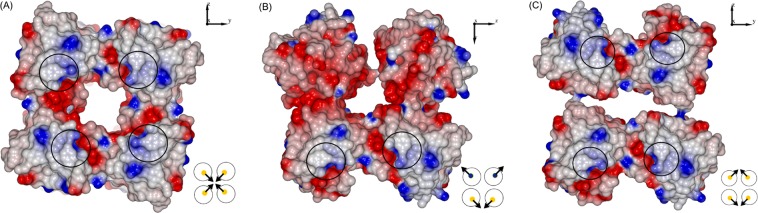
Surface Electrostatic Charge Distribution of Gh–TDH Structure. (**A**) The electrostatic molecular surface of the Oligomer–I with positive and negative electrostatic potentials are colored blue and red, respectively. The position of *N*–terminal residue Gly^12^ was marked with black circles. (**B**) The electrostatic molecular surface of the Oligomer–II. The loosely closure on top edge of the tetramer was made by one H–bond between residue Lys^97^ and Gln^104^. (**C**) The electrostatic molecular surface of the Oligomer–III. The closure on either left or right side of the tetramer was also made by one H–bond between residue Lys^97^ and Gln^104^.

Analysis of the solved two Gh–TDH structures showed that the proteins crystallized in the same *P*2_1_2_1_2 space group setting but in two different crystal packing patterns as shown in Fig. 1C,D. Remarkably, both structures had essentially the same unit cell dimensions of a = 105.3, b = 112.6, c = 60.8 Å, and the same content of four monomers per asymmetric unit in contrast to one monomer found in the homologous Vp–TDH (Supplemental Table 1)^23^. Gh–TDH shows solvent content and the Matthews coefficient V_m_ of 49.27% and 2.4 Å^3^/Da, respectively, whereas those of 44.41% and 2.2 Å^3^/Da were observed for Vp–TDH^23^. Several lines of biophysical studies and biochemical data have demonstrated that Vp–TDH in solution is tetrameric with *C*_4_ symmetry, although crystallographic analysis showed the asymmetric unit to contain a Vp–TDH protomer^22,23^. The Gh–TDH Oligomer–I structure (Fig. 1C) revealed the same tetramer oligomerization arrangement as for Vp–TDH tetramer. The topology of the two TDH tetrameric structures was almost identical, with the best rms difference of 0.66 Å for 616 C_α_ atoms (Supplemental Fig. 1). Moreover, two additional tetrameric forms, designated as Gh–TDH Oligomer–II and –III, were also found to have the same crystal packing in both Gh–TDH structures (Fig. 1C,D). In contrast to the *C*_4_ symmetry found in Gh–TDH Oligomer–I and Vp–TDH tetramer, the arrangement of the four Gh–TDH protomers in Oligomer–II was asymmetric; two monomers were packed in the same way as that of the Oligomer–I structure with the same orientation, whereas the other two monomers were packed with the dimer interface pointing outward and in opposite orientation to the formerly formed dimeric unit. The Gh–TDH Oligomer–III was formed by packing two of the same dimeric units found in Oligomer–I and –II in two–fold symmetry. All three types of Gh–TDH tetramers obtained from two different crystal packing patterns were consistently superimposable and shared a common motif of a dimeric unit consistent with that of the Vp–TDH tetramer, with the exception of Gh–TDH Oligomer–II and –III, where the facet of the dimeric unit originally facing the pore was turned 180° away to face outward to the solution (Fig. 2 lower schematic illustrations).

### Plausible multiple oligomerization interfaces of the Gh–TDH protomer

**Figure 3:**
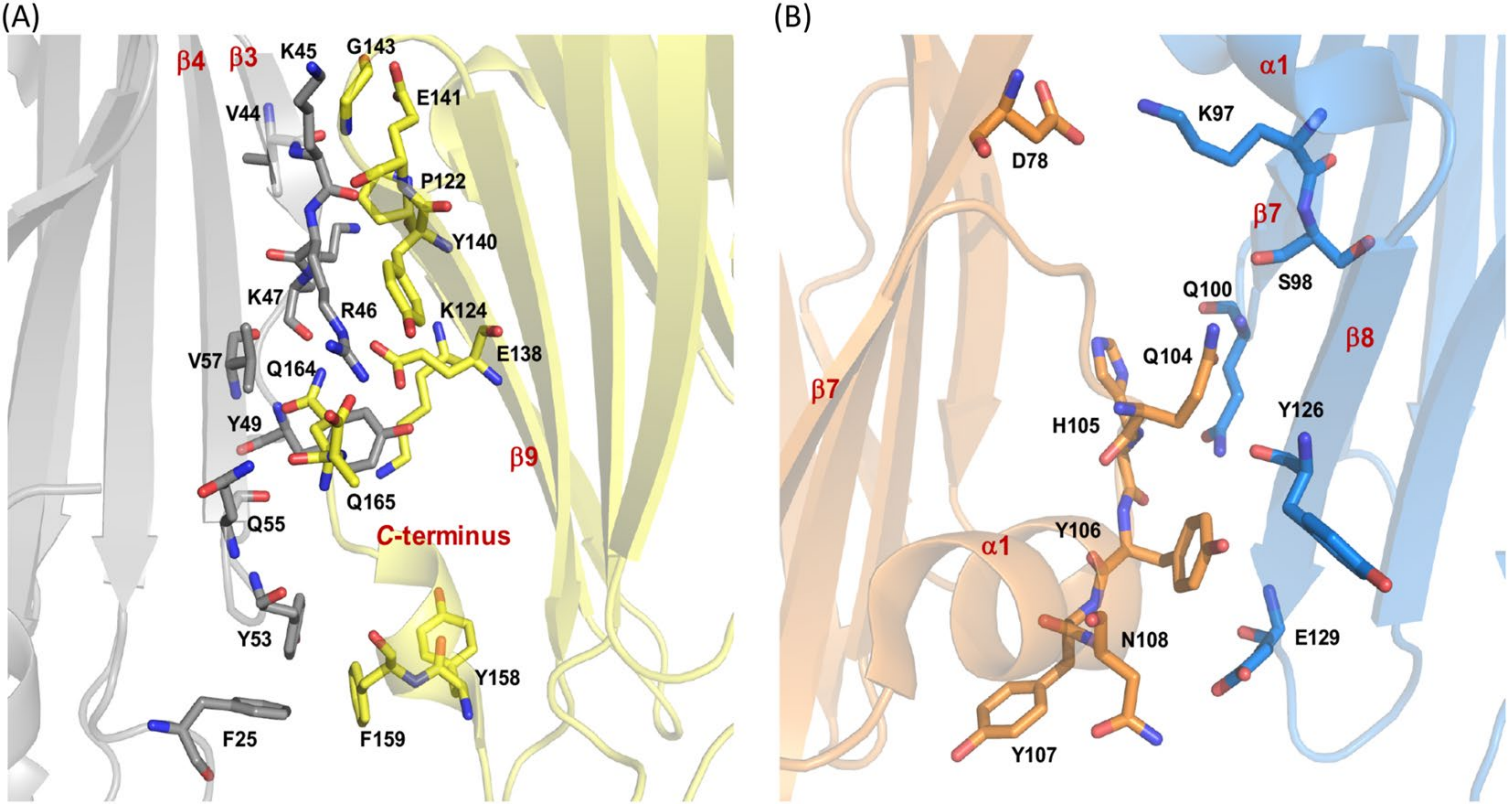
The Heterologous Interactions Involved in the Formation of Gh–TDH Oligomers. (**A**) Gh–TDH Oligomer–I. The two Gh–TDH protomers were colored in grey and yellow, respectively. The residue Arg^46^, Glu^138^, and Gln^164^ were involved in an intricate network of H–bonds in the protomer–protomer interface. (**B**) Gh–TDH Oligomer–II. The two Gh–TDH protomers were colored in orange and blue, respectively.

A close examination of three Gh–TDH tetramer structures revealed possible multiple regions in the Gh–TDH protomer for oligomerization in addition to that previously found in Vp–TDH structure. In the Gh–TDH Oligomer–I, residues mainly from two regions of β3–β4 and β9 and *C*–terminus were involved in the formation of the tetramer (Fig. 3A and Supplemental Fig. 2A,B). Except for the shape complementary, the protomer–protomer interactions involved 12 H–bonds and 4 salt bridges contributed from main– or/and side–chain atoms of residue Lys^45^, Arg^46^, Lys^47^, Tyr^49^, Gln^55^, and Val^57^ on β3–β4 corresponding to residue Glu^138^, Glu^141^, Gly^143^, Gln^164^, and Gln^165^(modeled as Ala) on the β9 and *C*–terminus region (Fig. 3A). Further 5–9 of hydrophobic, aromatic–aromatic, and cation–π interactions involving residues Phe^25^, Val^44^, Arg^46^, Tyr^49^, and Tyr^53^, to residues Pro^122^, Lys^124^, Tyr^140^, Tyr^158^, and Phe^159^, respectively, were located on top and bottom of the oligomerization interface (Fig. 3A). Especially, the residue Arg^46^ stood out and used its N_ε_, N_η1_ and N_η2_ atom of the guanidium group to form H–bonds with the side chain carboxyl group of Glu^138^, main chain oxygen atom of Gln^165^, and both the *C*–terminal carboxyl group and the main chain oxygen atom of Gln^164^ in the adjacent protomer. Moreover, the main chain nitrogen and/or oxygen atom and carboxylate group O_ε1_ and N_ε2_ atoms of Gln^164^ and Gln^165^, respectively, were also within H–bonding distance to residues Lys^47^, Tyr^49^, Gln^55^, and Val^57^ in the adjacent protomer. PISA analysis of Gh–TDH revealed that the total solvent accessible area of the Oligomer–I is 28567 Å^2^ and the tetrameric interface buries ~2649 Å^2^ of the accessible surface area (ASA) with 662 Å^2^ for the average protomer-protomer interface, supporting the formation of a stable tetramer^35^.

Three types of oligomerization interfaces were found in the Gh–TDH Oligomer–II since four protomers were packed asymmetrically. This tetramer contained a common dimeric unit, which was in opposite orientation as compared to that in Oligomer–I but exhibited essentially the same types of interactions as described earlier (Fig. 3A). However, the other two protomers were packed against the dimeric unit in reverse orientation and with their original dimer oligomerization interface facing outward, which in turn exposed the residues on the β7–α1–β8 region asymmetrically with respect to the same region presented by the dimeric unit. Figure 2B shows that residues Lys^97^, Ser^98^, Gln^100^, Tyr^126^, Glu^129^ on β7 and β8 from one side of the dimeric unit form mainly main chain to side chain H–bonds with residues Asp^78^, Gln^104^, His^105^, Tyr^106^, and Asn^108^ on the turn region (between β7 and α1 helix) close to the *N*–terminus of α1 in the adjacent up–side–down Gh–TDH protomer. Only one hydrophobic interaction between residue Tyr^126^ and Tyr^106^ was noticed. The interface on the other side of the dimeric unit presented same types of interactions but in opposite orientation and sequence, where the same residues on β7 and β8 in the other up–side–down Gh–TDH protomer interacted with the same residues on the turn region before α1 from the other side of the dimeric unit. The asymmetric arrangement of four Gh–TDH protomers in the Oligomer–II gave little interaction, only a H–bond between residues Lys^97^ and Gln^104^ (Supplemental Fig. 2A,C), between the two up–side–down protomers and yielded a fairly open region on one side of the pore forming tetramer. The total solvent accessible area of the Oligomer–II was 29643 Å^2^ and the average three types of protomer–protomer interfaces were 659, 441 and 42 Å^2^, respectively.

In Gh–TDH Oligomer–III, two dimeric units were packed against each other in opposite orientation resulting in a twofold symmetry as compared to the symmetric arrangement of the two dimer motifs in Oligomer–I. The interactions within the interface of these two dimeric motifs were essentially the same as previously described except for an additional H–bond formed between the amine nitrogen (N_ζ_) of Lys^47^ and the main chain oxygen atom in residue Pro^122^. However, the interaction, which was also asymmetric, between the two dimeric motifs was limited to a H–bond between residue Lys^97^ and Gln^104^ (Supplemental Fig. 2A,D) with a distance of 2.89 and 3.99 Å, respectively. The total solvent accessible area of the Oligomer–III was 29873 Å^2^ and the average two types of protomer-protomer interface were 632 and 43 Å^2^, respectively. In summary, the much less of total buried surfaces and interface interactions between protomers in Oligomer II and III than that of Oligomer–I suggest that Oligomer–I contributes to the major oligomerization. In parallel, analysis of the surface electrostatic charge distribution of Gh–TDH tetramers showed that all types were hydrophilic in nature with extended positive– and negative–charged patches throughout the structure (Fig. 2). The pores of this pore-forming Gh–TDH toxin were different in shape. In Oligomer–I, the pore was comparable with that of Vp–TDH tetramer with a diameter of 17 Å and approximately 50 Å in depth; In Oligomer–II, the pore was smaller in size with a diameter of ~13 Å and not fully closed on one side of the tetramer; In Oligomer–III, the pore was elongated and narrow in shape with dimension of ~36 by ~18 Å and with additional two sides in the tetramer structure accessible to aqueous solution.

### Biochemical studies of Gh–TDH mutants involved in oligomerization

**Figure 4:**
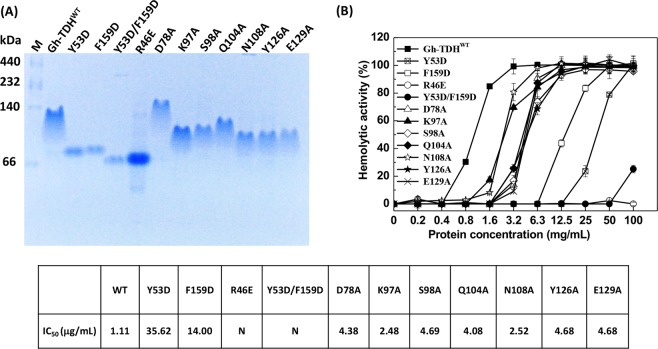
Gel Electrophoresis Analysis and Hemolytic Activity Assay of Gh–TDH^WT^ and Gh–TDH^mut^ Proteins. (**A**) Coomassie blue–stained native–PAGE of Gh–TDHs. (**B**) Hemolytic activity of Gh–TDH mutants putatively involved in protomer-protomer or membrane interactions. Hemolytic activity was assessed by measuring the absorbance at 540 nm at room temperature and compared with that of 0.1% Triton X–100–treated rabbit erythrocytes. The mean ± S.D. was determined from three independent measurements and shown as bars on data points.

To characterize the effects of protomer–protomer interface interactions on oligomerization and hemolytic activity, a series of mutants (R46E, Y53D, F159D, Y53D/F159D, D78A, K97A, S98A, Q104A, N108A, Y126A, and E129A) were constructed (Fig. 3 and Supplemental Fig. 2). Specifically, the basic Arg^46^ was mutated to acidic Glu^46^ to investigate the electrostatic effect, whereas the hydrophobic aromatic Tyr^53^ and Phe^159^ were mutated to acidic Asp^53^ and Asp^159^ to investigate the aromatic–aromatic interactions. In parallel, Asp^78^, Lys^97^, Ser^98^, Gln^104^, Asn^108^, Tyr^126^, and Glu^129^ were mutated to alanine to determine the secondary structure effects. All mutants were purified as the Gh–TDH^WT^ and the purity and oligomerization state were analyzed using both native– and SDS–PAGE. The native electrophoretic mobility results showed that mutants Y53D and F159D acted as dimers, whereas the R46E and Y53D/F159D acted as monomers (Fig. 4A). Consistent with the observation is that the analytical ultracentrifugation analysis (AUC) results were 33.09 ± 0.07 and 28.28 ± 0.15 kDa for the Y53D and F159D mutants as dimers; 21.88 ± 0.05 and 18.86 ± 0.007 kDa for the Y53D/F159D double mutant and R46E mutant as monomers, respectively, in contrast to 74.54 ± 0.007 kDa for Gh-TDH^WT^ tetramer (Table S2 and Fig. S6). The monomeric electrophoretic mobility of R46E mutated result supports that Oligomer–I is the major tetrameric form in solution where the salt–bridge between Arg^46^ and Glu^138^ contributes key interactions between four protomers (Fig. 3A). Asp^78^ and Lys^97^ form a salt–bridge in Oligomer–II (Fig. 3B); however, the D78A mutant migrated similarly as the wild–type TDH on native–PAGE (Fig. 4A), indicating that D78A mutant is still a tetramer. This result revealed that losing salt–bridge between Asp^78^ and Lys^97^ is unable to disrupt the tetramer formation and suggested that Oligomer–II is not a major oligomer form in solution. In addition, the K97A, S98A, Q104A, N108A, Y126A, and E129A mutants behaved as multimers but migrated differently than the wild–type Gh-TDH on native–PAGE (Fig. 4A).

Similarly, disrupting Gh–TDH tetramer formation would severely hamper toxin hemolytic activity as shown in Fig. 4B. Tyr^53^ and Phe^159^ form π–stacking interactions between Gh–TDH protomers (Supplemental Fig. 3). The mutant Y53D and F159D resulted in 20– and 10–fold decreases in activity, respectively, whereas Y53D/F159D double mutants resulted in a ~200–fold (15.8 ± 4.9%) drop in activity, and the R46E mutant resulted in null activity (Fig. 4B). The hemolytic activity of mutants D78A, K97A, S98A, N108A, and Y126A in multimeric forms was approximately 1~4 fold decreases than that observed for Gh–TDH^WT^.

### Putative residues of Gh–TDH protomer involved in membrane interaction

The DALI structural database search for homologous proteins revealed that Gh–TDH shares similar structural fold with those of PFT family proteins, including TDH from *V. parahaemolyticus*, equinatoxin II (Eqt II) from *Actina equina*^36^, sticholysin II (Stn II) from *Stoichactis helianthus*^37^, and lectins (XCL) from *Xerocomus chrysenteron* and *Agaricus bisporus*^38^, despite less than 15% amino acid sequence identities. Multiple sequences alignment using *ClustalW* with standard parameter did not reveal matches of structurally equivalent residues. These proteins exhibit similar secondary structures that are rich in β–sheets and form a tightly folded β–sandwich core with an α–helix on each side. Among them, Eqt II is a water–soluble transmembrane solute transporter which oligomerizes 3 to 4 monomers to create a cation–selective pore in model lipid bilayer and natural membranes^36^. Stn II does not contain any cysteine residues, but contains a large number of non-polar amino acids and has a highly alkaline isoelectric point (pI> 9.5)^37^. Both toxins, Eqt II and Stn II, form pores in 2–nm diameter pores composed of four monomers. XCL is a carbohydrate–binding protein that functions to disrupt cell–substrate adhesion via the formation of a tetrameric assembly with a very large cavity which subsequently blocks cell proliferation^38^. Three structural differences among these toxins could be identified where Gh–TDH carries an 11 residue *N*–terminal extension in contrast to Eqt II, Stn II and XCL carry a 31, 31, and no residue *N*–terminal extension, respectively. Both Gh–TDH and XCL contain a β3 strand, whereas a long loop of nine residues connecting the strands β2 and β4 was observed at the corresponding region in Eqt II and Stn II. Previous studies have shown that some of the tryptophan and tyrosine residues of membrane proteins are located at the water–lipid interface and participate in the formation of pores^39–42^. In Eqt II, there are at least two steps involved in the pore formation mechanism: initial membrane recognition and binding of surface exposed aromatic amino acids to membrane^43–45^; and subsequent transfer of the amphiphilic *N*–terminal helix into the lipid, leading to the formation of a functional pore^46^. A cluster of aromatic hydrophobic patch around the top of strand β2 (Phe^38^ and Trp^39^), the edge of strand β5 (Trp^65^), the loop between strands β6 and β7 (Tyr87, Phe^91^, and Phe^95^), and the helix α1 (Tyr^106^, Tyr^107^, and Phe^113^) was identified in Gh–TDH and Eqt II structures very likely to be the membrane binding sites (Fig. 1B)^47^.

**Figure 5:**
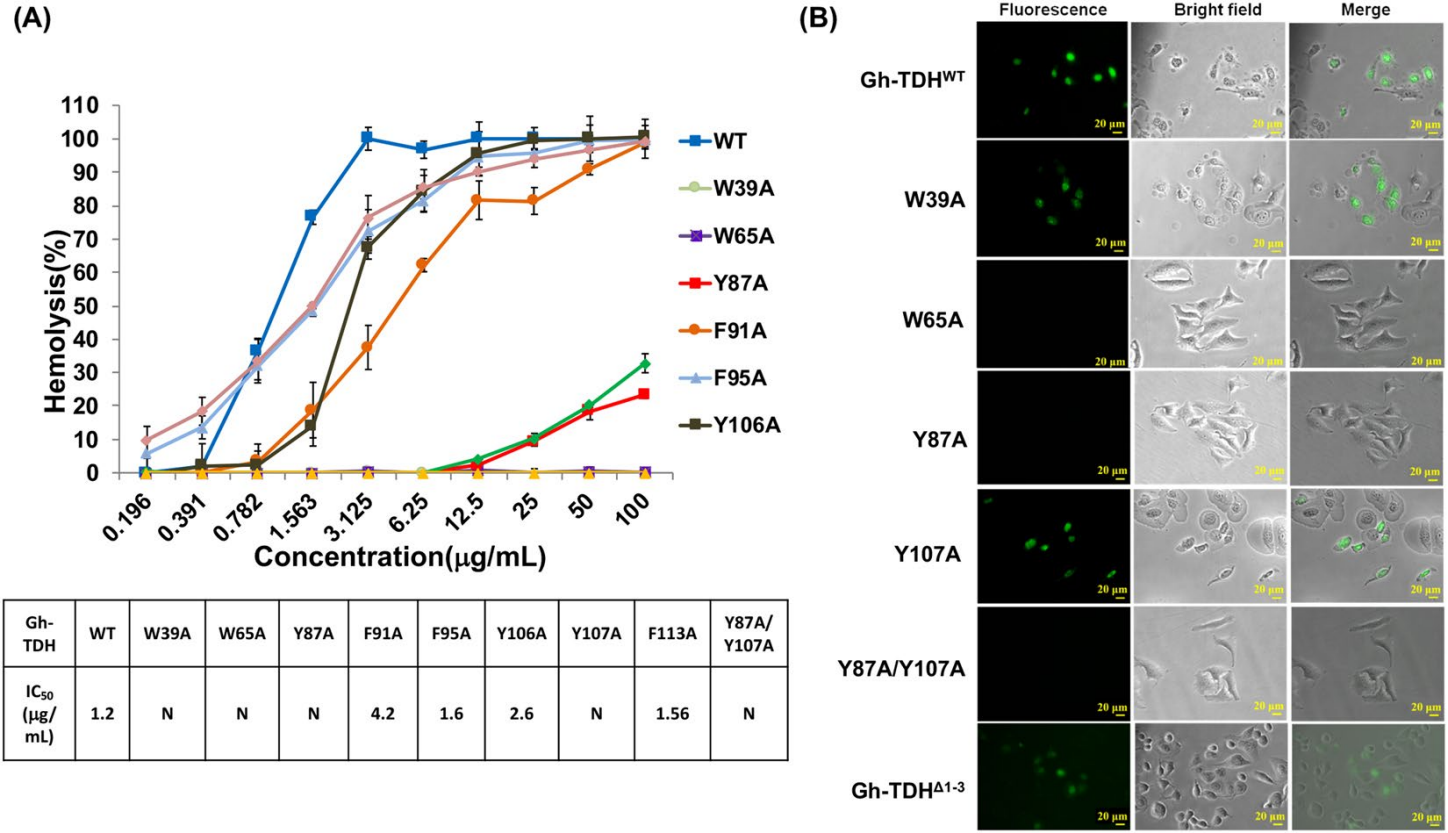
Hemolytic Activity and Fluorescence Microscopy Analyses of Wild–Type and Mutated Gh–TDHs. (**A**) Hemolytic activity of wild–type and mutated Gh–TDHs. (**B**) Fluorescence microscopy scan of HeLa cells for membrane binding and cell morphology changes after treated with FITC–conjugated wild–type and mutants of Gh–TDH proteins. The horizontal bars on the lower right-hand corner denote a scale of 20 μm.

A set of nine alanine-scanning mutants, W39A, W65A, Y87A, F91A, F95A, Y106A, Y107A, F113A, Y87A/Y107A (Fig. 1B) and the *N*–terminus truncated Gh–TDH^Δ1–3^, were used to screen for essential residues in membrane interaction^39–42,47^. The hemolytic activity assay results showed F91A mutation results in a four–fold reduction of hemolytic activity, and F95A, Y106A, and F113A mutants showed indistinguishable hemolytic activity from the wild type toxin; W39A, W65A, Y87A/Y107A, and Gh–TDH^Δ1–3^ mutants show no detectable activity. The hemolytic activity of Y87A and Y107A mutants dropped to only 24.4 ± 1.2% and 32.8 ± 2.8%, respectively, at the tested concentration of 100 μg/mL (Fig. 5A). We next assayed the membrane binding abilities of W39A, W65A, Y87A, Y107A, Y87A/Y107A and Gh–TDH^Δ1–3^ mutant using flow cytometry (Supplemental Fig. 3). The results indicated positive erythrocytes membrane binding for Gh–TDH^WT^, W39A, Y107A and Gh–TDH^Δ1–3^ mutants, whereas no change in fluorescence signal were observed for other Gh–TDH mutants. HeLa cell morphology analysis showed that W39A and Y107A mutants at concentrations of 10 μg/mL exhibit apparent membrane bubbling and cell detachment together with detectable fluorescence signals, whereas no effect was observed for W65A, Y87A, and Y87A/Y107A mutants and for BSA negative-control protein (Fig. 5B). Interestingly, W39A and Y107A mutants showed no or reduced hemolytic activity but both had apparent membrane binding activity for erythrocytes and HeLa cells as well as cytotoxicity. These results suggested that these three Gh–TDH mutants retain the ability to bind erythrocytes although the hemolytic activities were completely lost or compromised, while other Gh–TDH mutants were deficient in both membrane binding and blood hemolysis activity.

## Discussion

Thermostable direct hemolysin from *Vibrio* species has been characterized as pore–forming toxin and its tetrameric form is indispensable for hemolysis. Using monoclonal antibodies to probe the functional domains of Vp–TDH, Takeshi *et al*. suggest the *N*–terminal 1–31 residues are involved in the binding of TDH to red blood cells^48^. In this study, we provided structural evidence of a pore–forming TDH in multiple oligomeric conformations. These determined Gh–TDH oligomer structures were not caused by crystal packing effects, since the results from two separate MAD data provided the same set of three consistent Gh–TDH tetrameric oligomers. Among these oligomers, Gh–TDH Oligomer–I superimposed well with the reconstituted Vp–TDH tetrameric structure (Supplemental Fig. 1). These chemically identical but crystallographically distinct Gh–TDH oligomers are unique because it is rare to encounter multiple conformations of the oligomer to the same order of magnitude, whereas multiple oligomerization of proteins from one form to the other, such as dimer or monomer to oligomer of higher order, is more common. One example is the *Helicobacter pylori* vacuolating cytotoxin (VacA), which oligomerizes in multiple conformations including hexamers, dodecamers, and tetradecamers^49,50^.

Previously, two separate studies on channel conductance of Vp–TDH in planar lipid bilayer identified three types of small, intermediate, and large channels, which imply that TDH may undergoes a conformational change or adopt several oligomeric states during hemolysis of the membrane^23,51,52^. By analyzing the topology of the channels in tetrameric Gh–TDH oligomers (Supplemental Fig. 4), we proposed Oligomer–I as an intermediate, –II as a small, and –III as a large channel, respectively. The central pore of all Gh–TDH oligomers are shaped like an asymmetric hourglass with grooves extended out from the center of the pore on both ends and a narrowing at the middle of the channel. Thus, it seemed intuitive to measure the size of the bottle–neck in the channel and the numbers of side–channel as a way to assess the ion permeability of each oligomer. The central pore was not fully closed by the surrounding four Gh–TDH protomers and had several loose side channels. In Oligomer–I, the opening dimension of the central pore was within the range of 5.5 to 7.0 Å. The narrow passage of four additional side channels with a radius of ~1.0 Å was impermeable for water molecules with an accessed diameter of 2.8 Å. In Oligomer–II, the central pore size was around 4.8–5.5 Å. The radii of two and three side channels on either side of the tetramer, respectively, were in the range of 1.0–1.5 Å, which also were too small for water permeability; whereas, two large side–channels located only on one side of the tetramer had radius dimensions around 3.0 Å. In Oligomer–III, the pore was shaped like a narrow gorge on both ends of the pore and the opening dimension was around 5.0–6.3 Å. Two additional side-channels located on opposite sides of the tetramer had radii dimensions of around 3.0 Å, which were larger than the diameter of water molecules for permeability. The TDH channel exhibits low and similar ion selectivity and permeability since the radius of the central pore of all Gh–TDH oligomers found here is approximately 5.5 Å.

The binding mode of TDH to the artificial trisialoganglioside-G_T1b_ receptor containing lipid membranes, characterized by Yanagihara *et al*. using transmission electron microscopy, shows that tetrameric TDH attached either diagonally with one protomer or horizontally with two protomers^23^. Whether the colloidal osmosis of the erythrocyte or other types of nucleated cells truly require a complete attachment of four toxin protomers to form a pore forming channel has not been shown with any concrete experimental evidence so far. The currently determined Gh–TDH tetrameric Oligomer–I, and especially Oligomer –II and –III structures containing at least one common dimer motif, were in agreement with both binding modes. This also suggests that the Gh–TDH dimer motif is the minimum unit capable of membrane binding and presumably hemolytic activity (Fig. 4). The various tetramers, Oligomer–I, –II, and –III, constructed in the dimer motifs, may provide different hemolytic activities. The remaining question would be whether the orientation of the *N*–terminal face of the dimeric motif is crucial for membrane binding since the orientation of the dimer motif was different in Gh–TDH oligomers. According to the small angle X–ray scattering study of the flexible *N*–terminus of Vp–TDH, the hydrophobic *N*–terminal region protrudes from the central pore and links the tetramer together^22^. Therefore, the orientation of the dimer motif may have little effect on the conformation of the flexible *N*–terminal 1–31 residues within the tetrameric architecture. These pleomorphic Gh–TDH tetramers were in dynamic oligomeric equilibrium in solution, a feature of all multimeric structures of varying oligomeric order. The polar nature judging from the analysis result of the interactions, at most 12 H–bonds and 4 salt–bridges in contrast to the 4 hydrophobic, 2 π–aromatic interactions and ~600 Å^2^ buried interface area of the Gh–TDH protomer-protomer interface suggest that the non-obligate Gh–TDH binds and dissociates *in vivo* to form multiple pleomorphic complexes. However, it would be difficult to know if these three types of Gh–TDH tetramers were of equal amounts in solution, since the position and nature of the equilibrium have yet to be determined experimentally.

The mechanism of the TDH’s unique Arrhenius effect has been characterized in detail and found to be tightly related to the fibrillogenicity of TDH^24^. TDH aggregates into β–strand-rich fibrils after heating at 60–70 °C and dissociates into an unfolded state after further heating at 80 °C or higher^24^. This reversible fibril aggregation phenomenon resembles amyloidogenicity of polypeptides yielding cross–β structures related to specific diseases like Alzheimer’s disease, amyloidosis, and prion-caused neurodegenerative diseases^24,53^. Although the amyloidogenic proteins vary without conserved sequences, the multiple Gh–TDH protomer-protomer interfaces as well as the heterologous assemblies of the Gh–TDH protomers in the oligomer structures could be used in modeling to understand the inter–subunit interactions of these amyloid proteins in their quaternary structure.

Pore formation of Gh–TDH is hypothesized to be a three–step process: initial recognition and interaction between the Gh–TDH molecule and the membrane, subsequent oligomerization of the 2–4 protomers for pore formation, and the post–insertion in erythrocyte hemolysis and cell cytolysis activities. Comparison of crystal structure between Gh–TDH and PFTs suggested that both the first 12 residues on the *N*–terminus and a patch of solvent–exposed aromatic residues are putative candidates for pore formation. It has been suggested that the *N*–terminal helix of Eqt II in *A. equina* is involved in lipid membrane interaction, although not as a requisite for binding^54,55^. Judging from the current determined crystal structure of Gh–TDH, one could safely assume the first 12 residues on the *N*–terminus had little effect in disrupting protein folding. Nevertheless, a *N*–terminus truncated Gh–TDH^Δ1–3^ mutant resulted in null hemolytic activity but had apparent binding affinity for erythrocytes analyzed by flow cytometry. This supports the notion of the functional role of the *N*–terminus in Gh–TDH for oligomer formation and hemolysis but not in the initial membrane interaction. In contrast, the solvent–exposed aromatic residues located near the water–lipid interface have been suggested to participate in the initial interaction with the lipid membrane^39–42,47^. We proposed to mutate tryptophan or tyrosine residues with functional homologues to that of Eqt II, melittin, and viral fusion peptides to characterize membrane interactions or pore forming abilities for toxin activity^47,54,56^. The results of alanine scanning mutagenesis suggested that W39A and W107A mutants retained lipid membrane binding capacity but lost their hemolytic activity, whereas W65A and Y87A mutants had both the membrane binding and the subsequent cell cytotoxicity abolished. Taken together, these findings suggested that Trp^65^ and Tyr^87^ residues mediate initial attachment of Gh–TDH to erythrocytes and cell membrane, whereas the *N*–terminal flexible helix and both the Trp^39^ and Tyr^107^ residues are related to post-insertion in Gh–TDH hemolytic and cytotoxic activities. Our results are consistent with a multi–step pore formation model of Gh–TDH, as described below: (1) Initial interaction of aromatic residues, which involve the residues Trp^65^ and Tyr^87^ at minimum, with lipids at the membrane surface; (2) Conformational changes of the toxin, including the *N*–terminal region, to assist with the insertion step which further leads to toxin oligomerization; (3) Post–binding process of hemolytic and cytotoxic activities which involve the residues Trp^39^ and Tyr^107^ at minimum.

Herein, a combination of structural and biochemical approaches was used to understand the structure-function relationship of Gh–TDH in membrane binding and hemolytic activity. Additionally, we presented structural evidence to show how a pore–forming bacterial toxin, Gh–TDH, breaks from the perfect closed, symmetrical oligomers of *C*_4_ cyclic symmetry to two other pleomorphic oligomers of the same order but with lower cyclic symmetry of *C*_2_ and *C*_1_. These three types of Gh–TDH tetramers could have the same potency in membrane binding and possibly hemolytic activity because all oligomers contained at least one common dimer motif. Evolutionarily, the dynamic nature of oligomerization of the released TDH toxins could quickly provide a wide diversity of functionally potent oligomers, instead of a single type of symmetrical oligomer, thus yielding the optimal state for a given functional niche and the advantage of better survival.

## Supplementary information

Supplementary Data for: Multiple Pleomorphic Tetramers of Thermostable Direct Hemolysin from Grimontia hollisae in Exerting Hemolysis and Membrane Binding
