# Supplementary material for: Multiple Pleomorphic Tetramers of Thermostable Direct Hemolysin from Grimontia hollisae in Exerting Hemolysis and Membrane Binding
Source: Sci Rep. 2019 Jul 8;9:9833. doi: 10.1038/s41598-019-46354-x (PMC6614540; doi:10.1038/s41598-019-46354-x)
Supplement: Supplementary file 1 — Supplementary Data for: Multiple Pleomorphic Tetramers of Thermostable Direct Hemolysin from Grimontia hollisae in Exerting Hemolysis and Membrane Binding [file 41598_2019_46354_MOESM1_ESM.pdf]

Supplementary Data for:

Multiple Pleomorphic Tetramers of Thermostable Direct Hemolysin from *Grimontia hollisae* in Exerting Hemolysis and Membrane Binding

Yu-Kuo Wang[a], Sheng-Cih Huang[a], Chin-Yuan Chang[a], Wan-Ting Huang[a], Man-Jun Liao[a], Bak-Sau Yip[c], Feng-Pai Chou[a], Thomas Tien-Hsiung Li[d]*, and Tung-Kung Wu[a,b]*

[a]Department of Biological Science and Technology, National Chiao Tung University, Hsin-Chu 30010, Taiwan, Republic of China

[b]Center for Emergent Functional Matter Science, National Chiao Tung University, 1001 Ta-Hsueh Rd., Hsinchu 30010, Taiwan, Republic of China

[c]Department of Neurology, National Taiwan University Hospital, Hsin-Chu, 30059, Taiwan, Republic of China

[d]Graduate Institute of Biochemistry, National Chung Hsing University, Taichung, 40227 Taiwan, Republic of China

*** To whom correspondence should be addressed:**

E-mail: tkwmll@mail.nctu.edu.tw (T. K. Wu)

Fax: +886-3-5725700

Tel: +886-3-5712121-56917

E-mail: lithomas@dragon.nchc.edu.tw (Thomas T. H. Li)

Fax: +886-4-22853487

Tel: +886-4-22840468

Fig. S1 Superposition of the overall structures of the TDH tetramers (Gh–TDH Oligomer–I) between *G. hollisae* (green, PDB entry 4WX3) and *V. parahaemolyticus* (red, PDB entry 3A57). The spheres colored in orange marked the position of the *N*-terminal Gly^{12} for each TDH monomer. The Vp–TDH tetramer were generated by applying crystallographic symmetries (Space group I_4) on the coordinates of the published Vp–TDH monomeric structure.

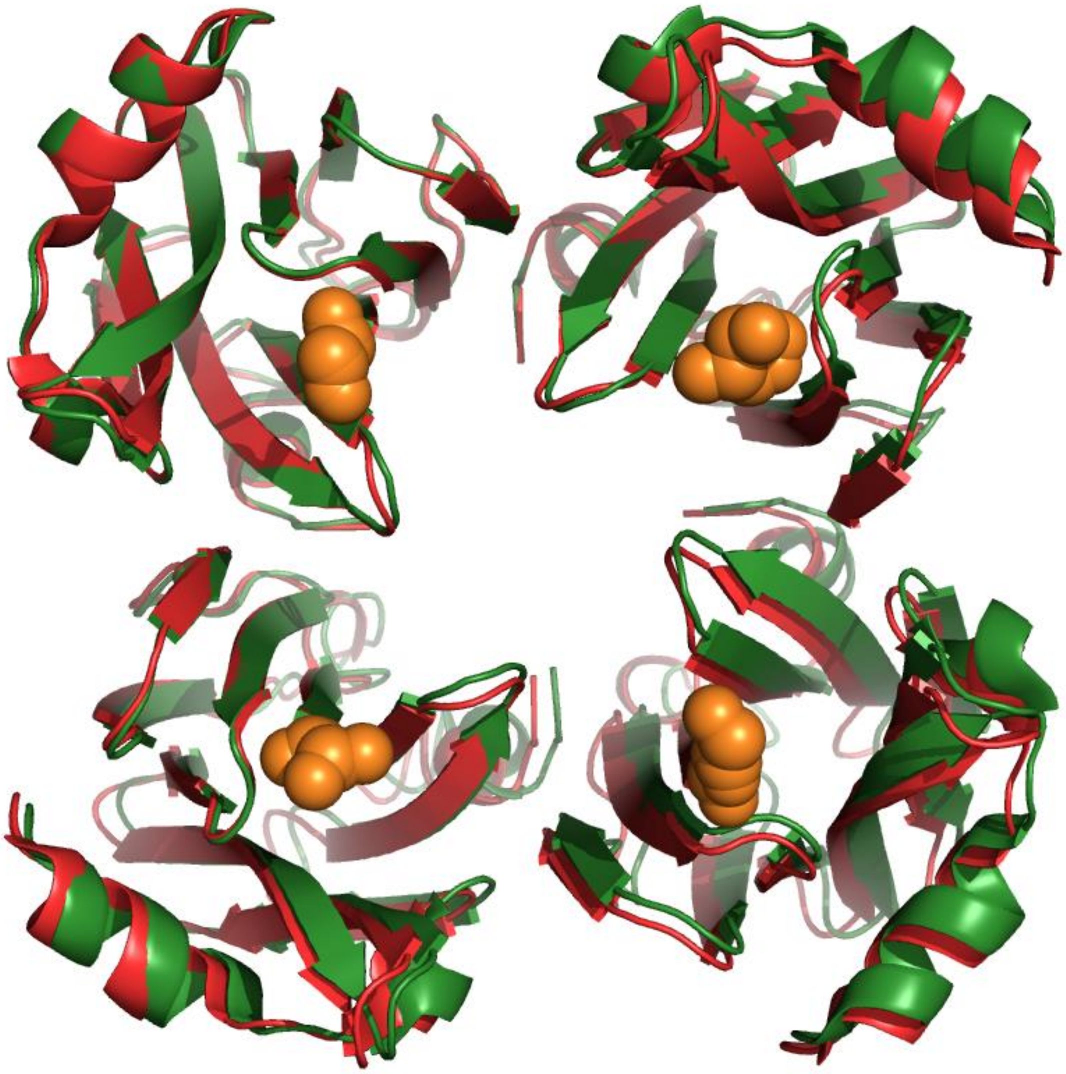

Fig. S2. Illustration of residues of Gh–TDH involved in protomer–protomer interactions. (A) The crystal packing pattern corresponds to the Gh–TDH–I oligomer, and protomer–protomer interactions in (B) Oligomer–I, (C) Oligomer–II, and (D) Oligomer–III.

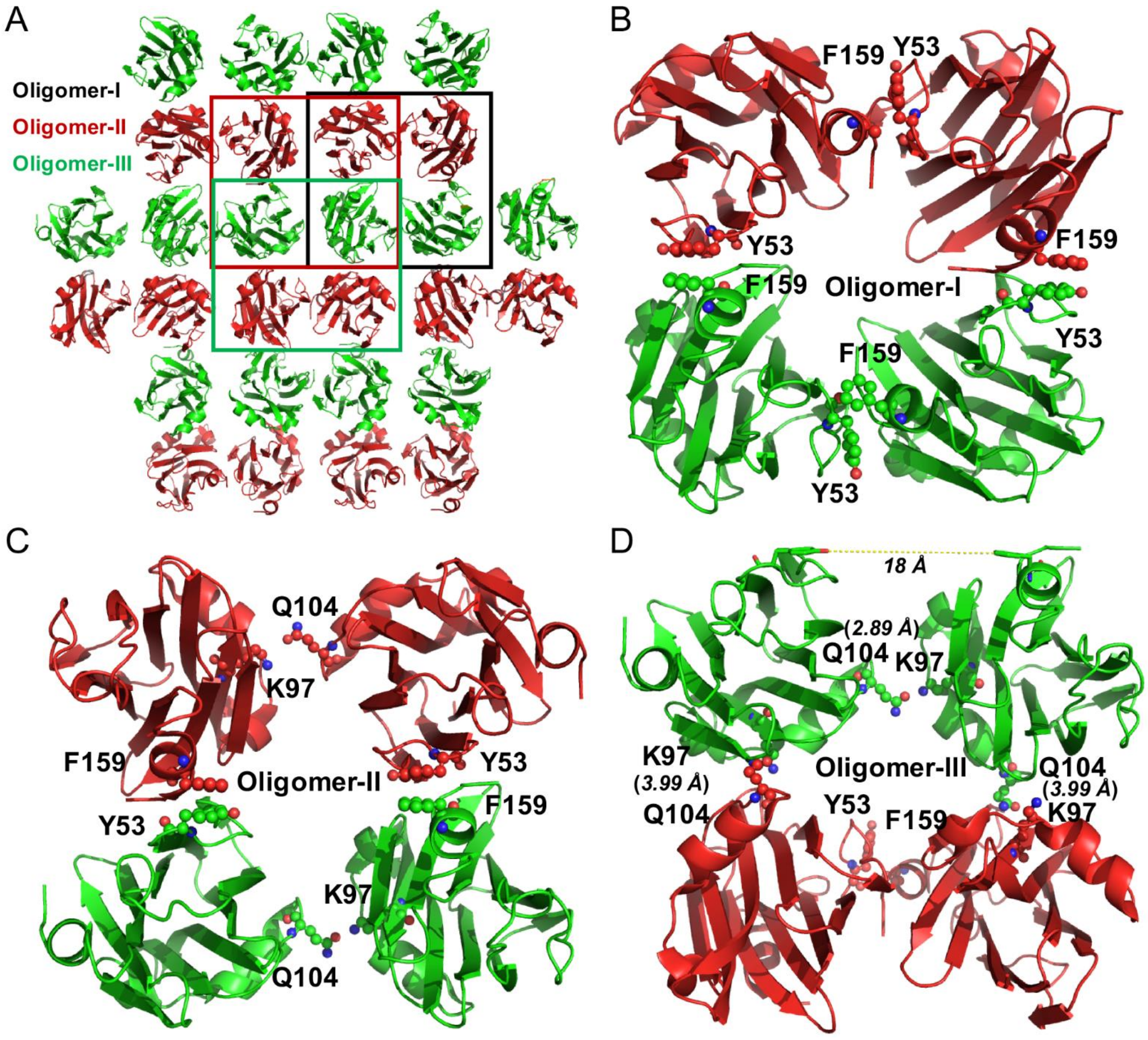

Fig. S3. Flow cytometry analysis of erythrocytes binding of wild–type and mutated Gh–TDHs.

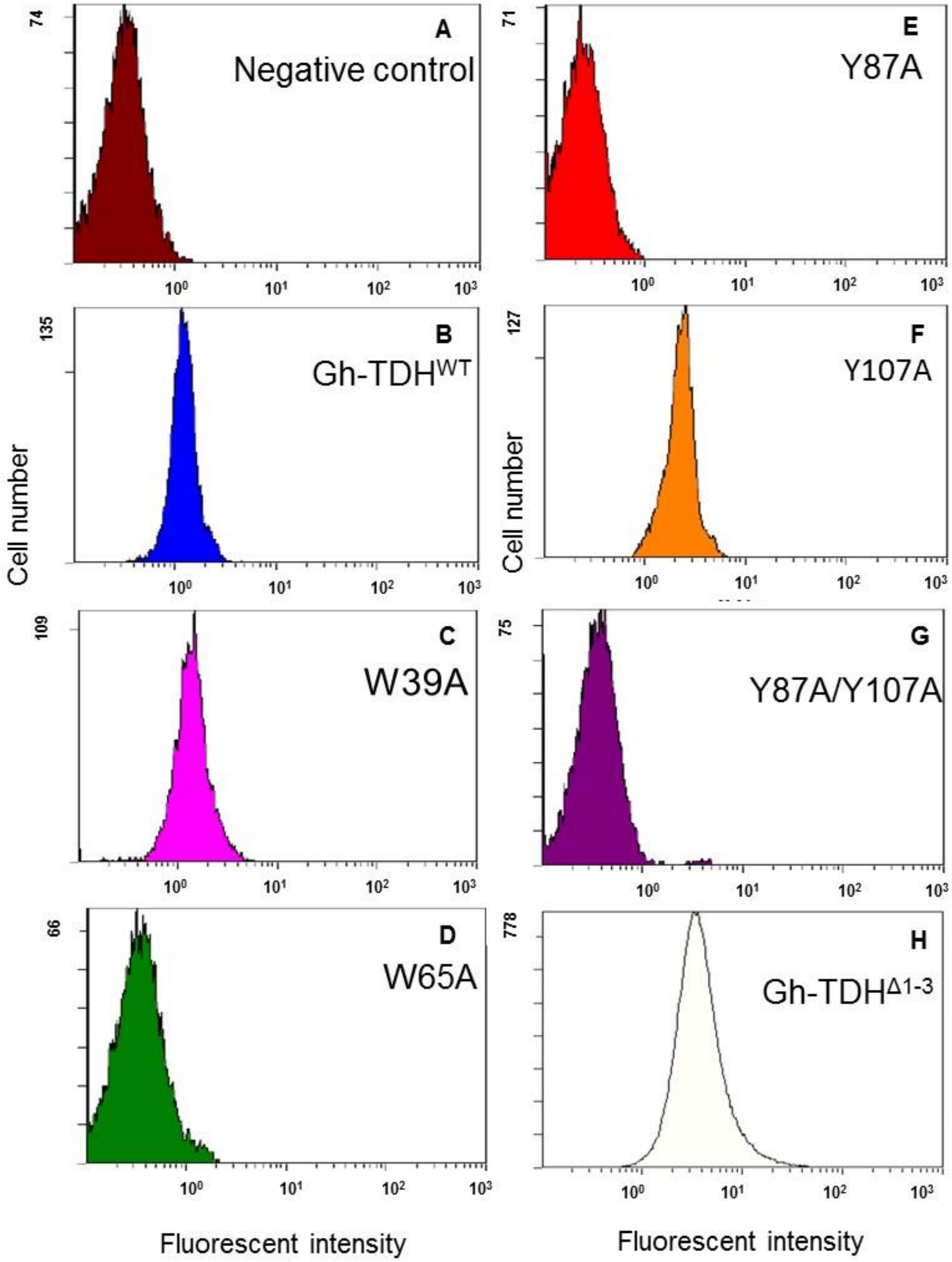

Fig. S4A. Illustrations of the analysis of the topology of Gh–TDH Oligomer–I, –II , and –III using program CAVER [50]. The narrowest part of the side–channels were marked with measurements.

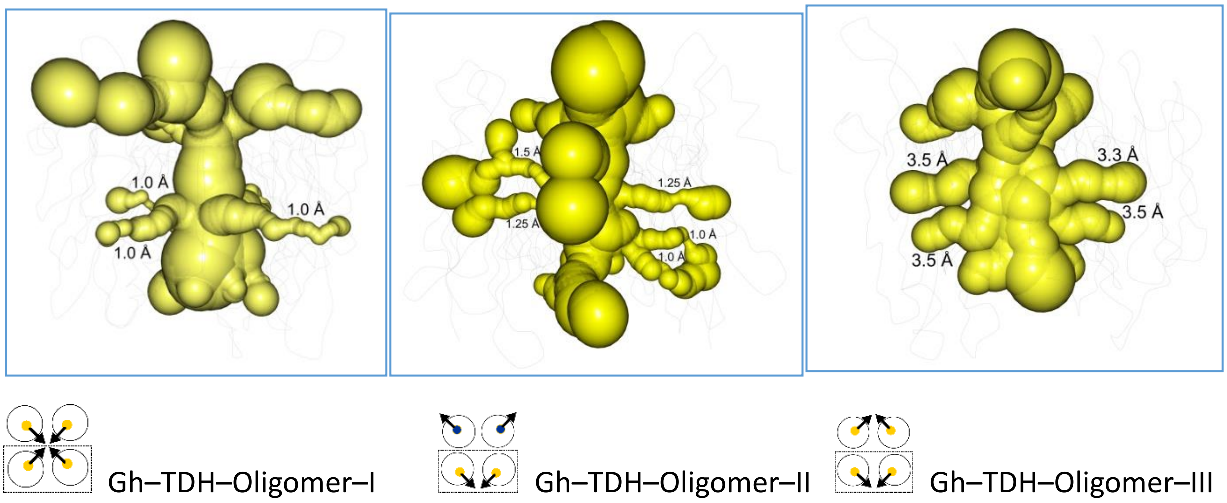

Fig. S4B. Top–view of Oligomer–I from the *N*–terminus direction and of Oligomer–II and –III.

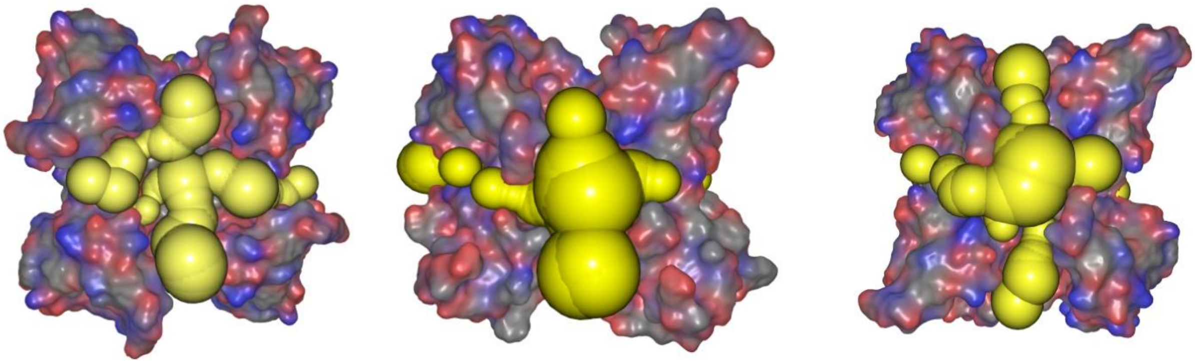

Figure S4C. Bottom–view of Gh–TDH Oligomer–I from the C–terminus direction and of Oligomer–II and –III.

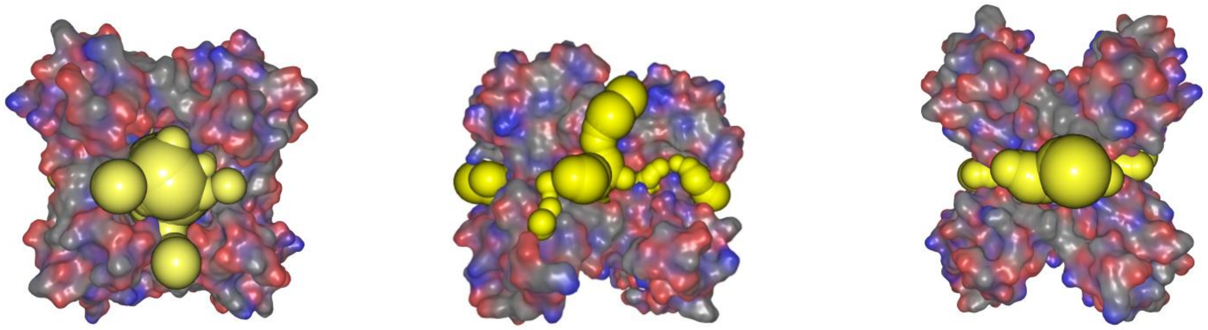

Fig. S5. Sequence alignment of Vp−TDH and Gh−TDH.

```
G.hollisae_TDH                 FELPSIPFPSPGSDEILFVVRDTTFNTKEPVNVKVSDFWTNRNVKRKPYK 50
V.parahaemolyticus_TDH2        FELPSVPFPAPGSDEILFVVRDTTFNTNAPVNVEVSDFWTNRNVKRKPYK 50
                               *****:***:*****************: ****:****************

G.hollisae_TDH                 DVYGQSVFTTSGSKWLTSYMTVSINNKDYTMAAVSGYKDGFSSVFVKSGQ 100
V.parahaemolyticus_TDH2        DVYGQSVFTTSGTKWLTSYMTVNINDKDYTMAAVSGYKHGHSAVFVKSDQ 100
                               ************:*********.**:************.*.*:*****.*

G.hollisae_TDH                 IQLQHYYNSVADFVGGDENSIPSKTYLDETPEYFVNVEAYESGSGNILVM 150
V.parahaemolyticus_TDH2        VQLQHSYDSVANFVGEDEDSIPSKMYLDETPEYFVNVEAYESGSGNILVM 150
                               :**** *:***:*** **:***** *************************

G.hollisae_TDH                 CISNKESYFECESQQ 165
V.parahaemolyticus_TDH2        CISNKESFFECKHQQ 165
                               *******:***: **
```

Fig. S6. Analytical ultracentrifugation analysis (AUC) results of Gh–TDH^{WT} and various Gh–TDH^{mut} proteins. (A) Gh–TDH^{WT}; (B) Gh–TDH^{Y53D}; (C) Gh–TDH^{F159D}; (D) Gh–TDH^{R46E}; (E) Gh–TDH$^{Y53D/F159D}$; (F) Gh–TDH^{D78A}; (G) Gh–TDH^{K97A}; (H) Gh–TDH^{N108A}.

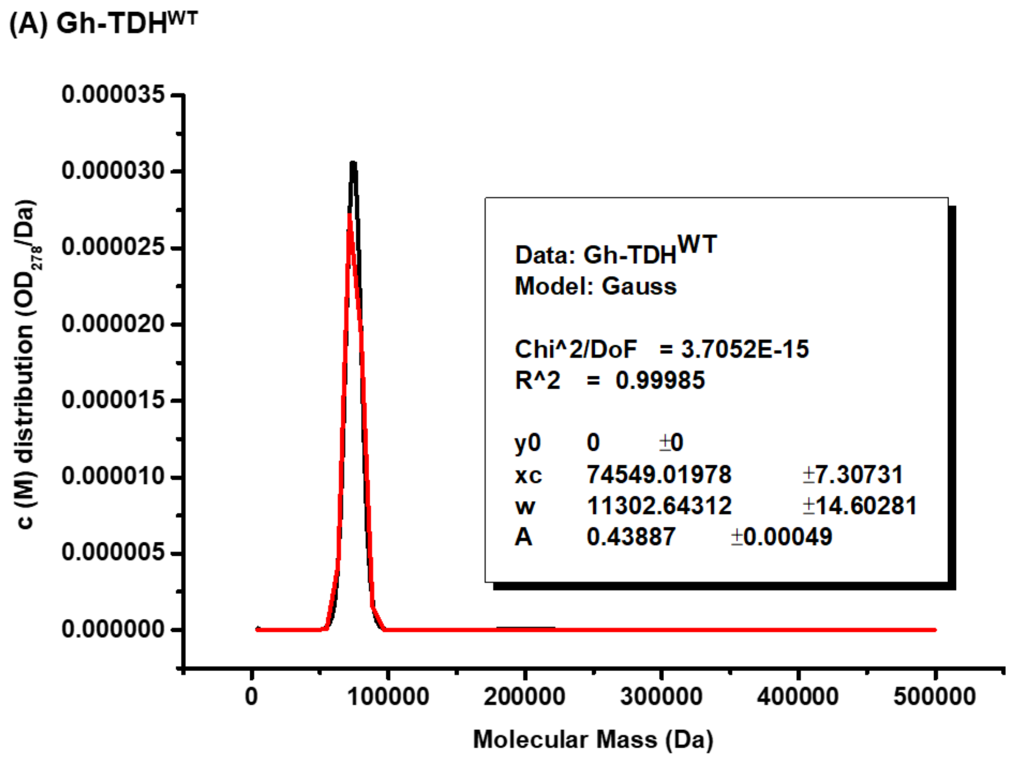

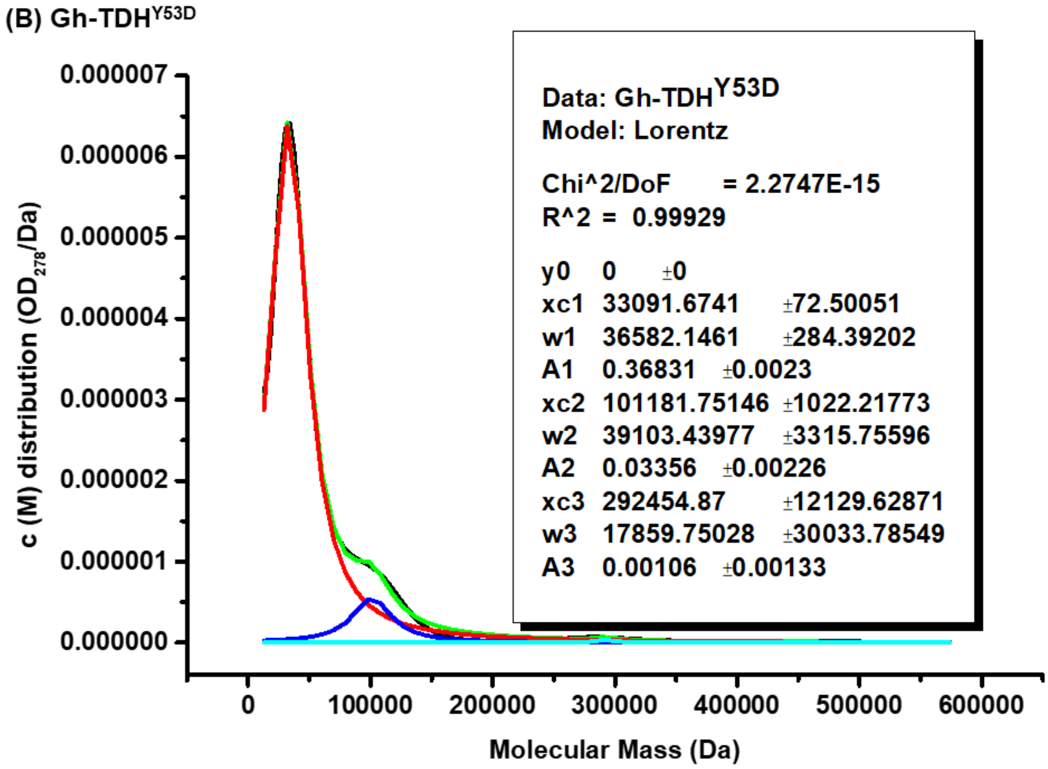

(C) Gh-TDH^{F159D}

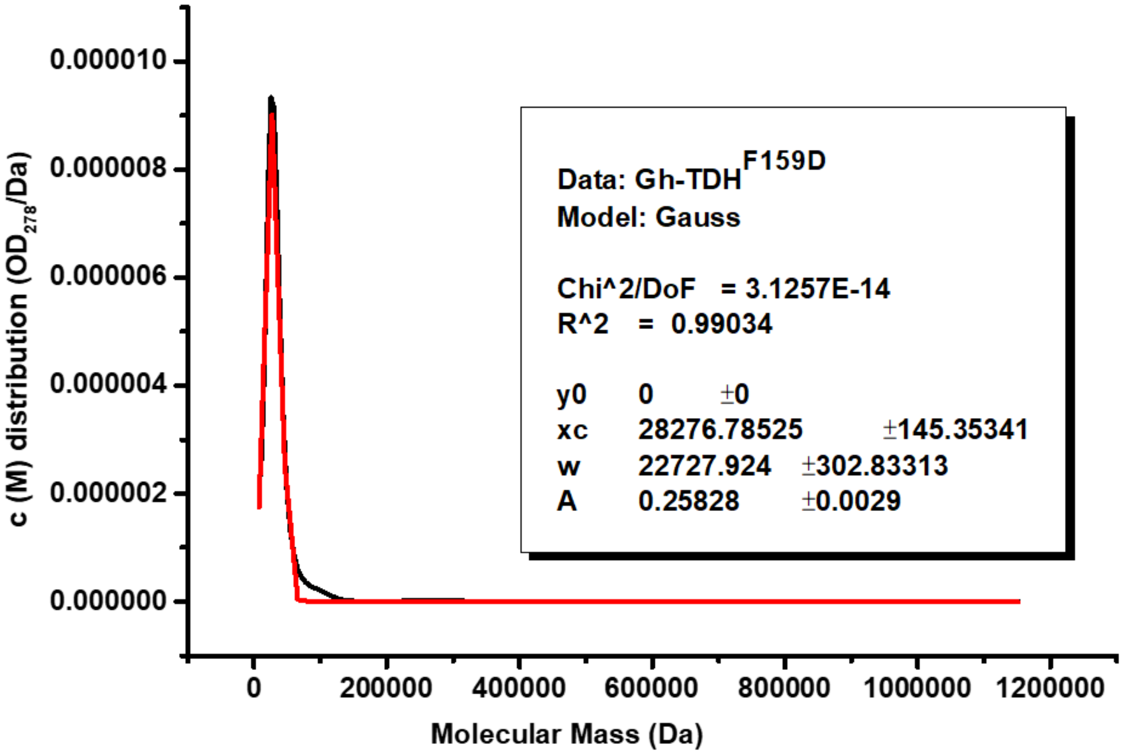

(D) Gh-TDH^{R46E}

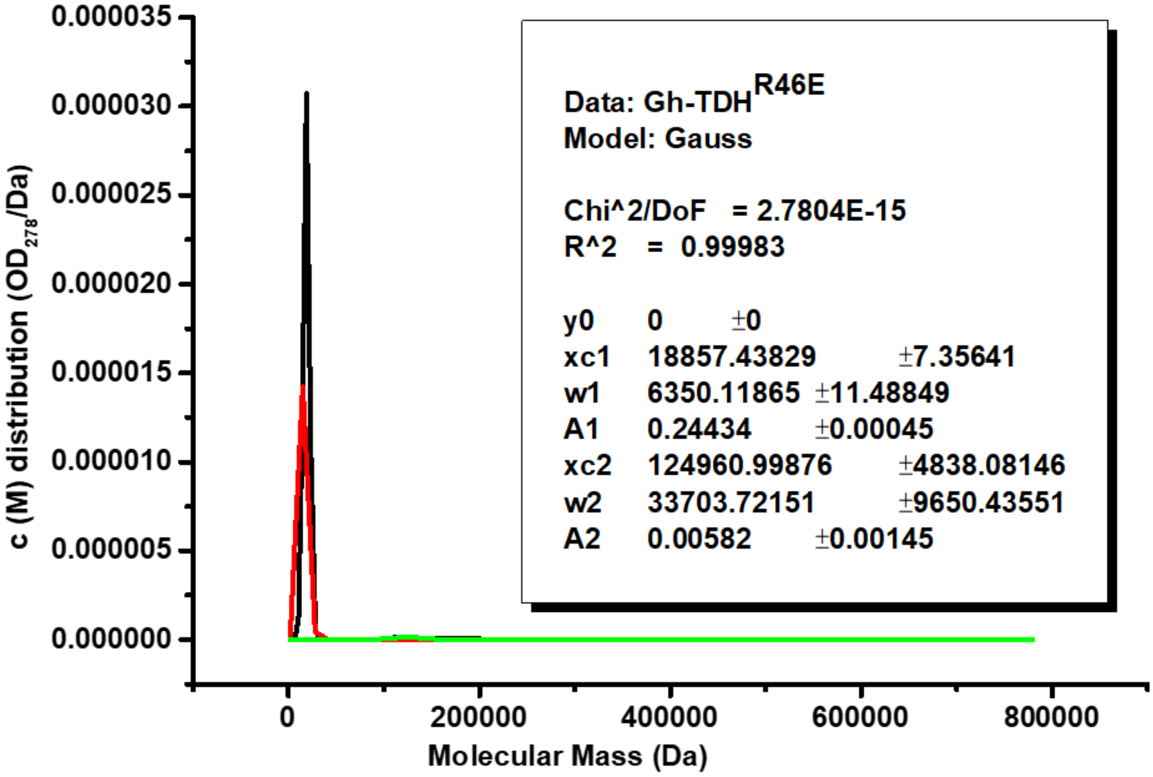

(E) Gh-TDH$^{Y53D/F159D}$

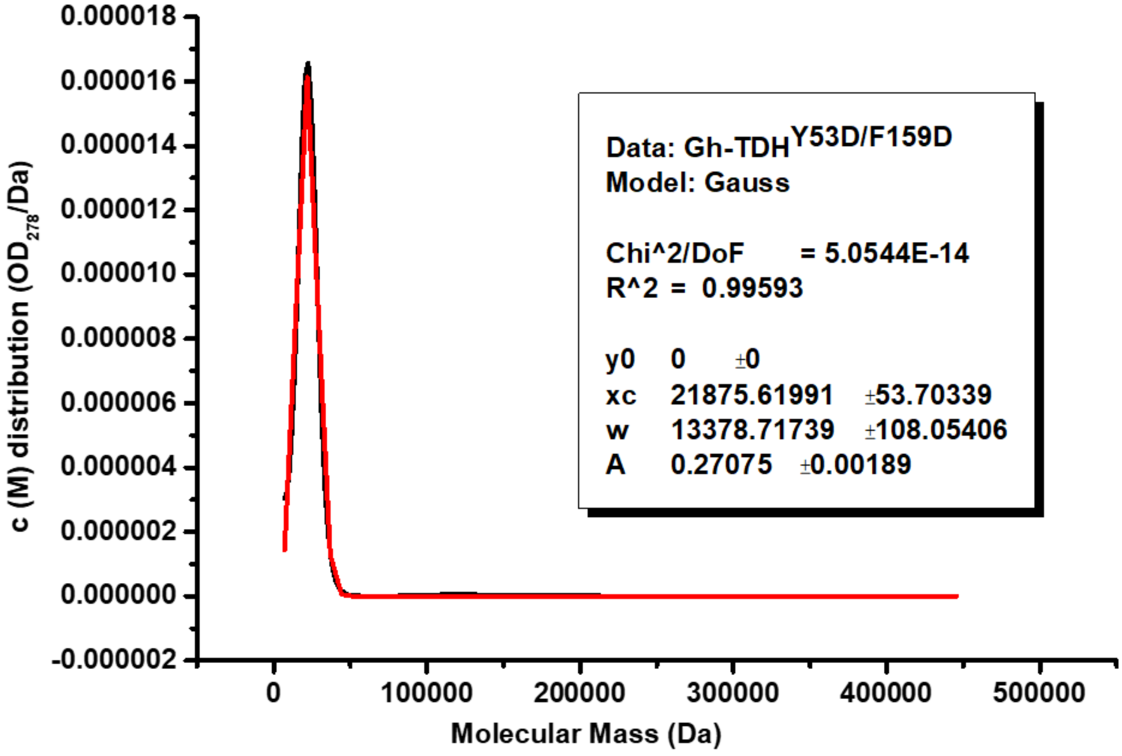

(F) Gh-TDH^{D78A}

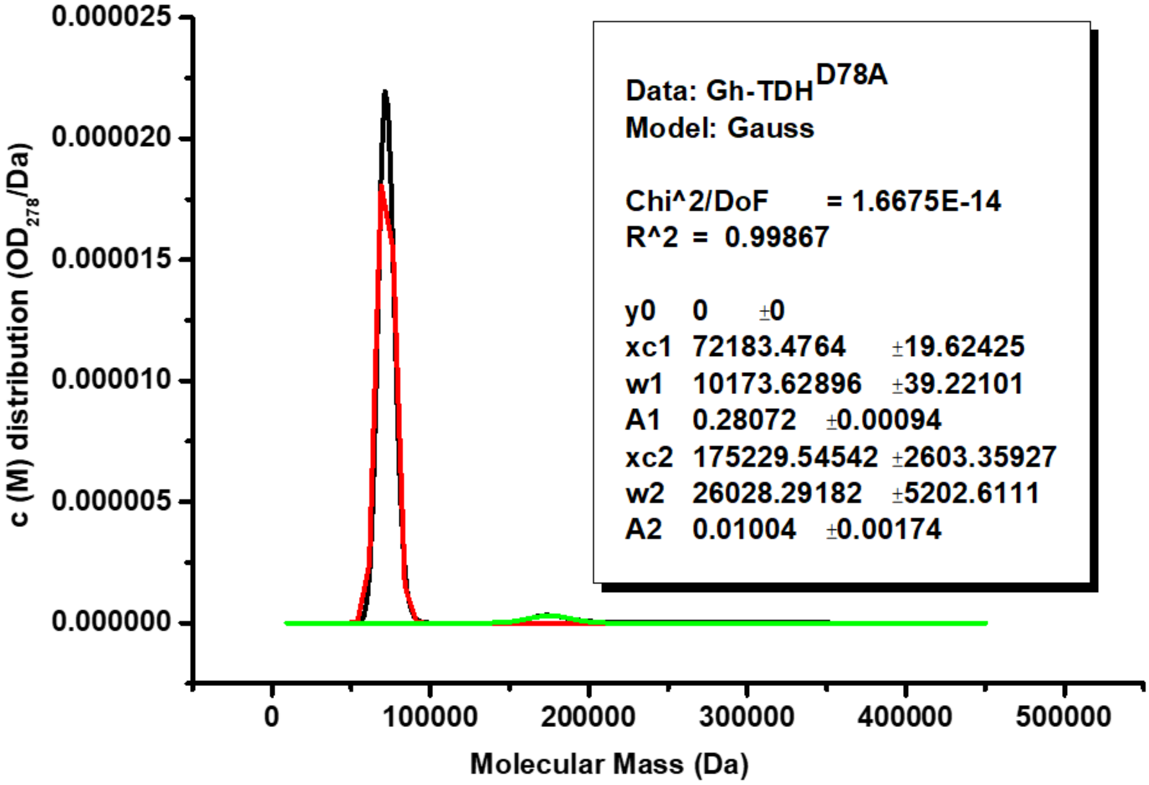

(G) Gh-TDH^{K97A}

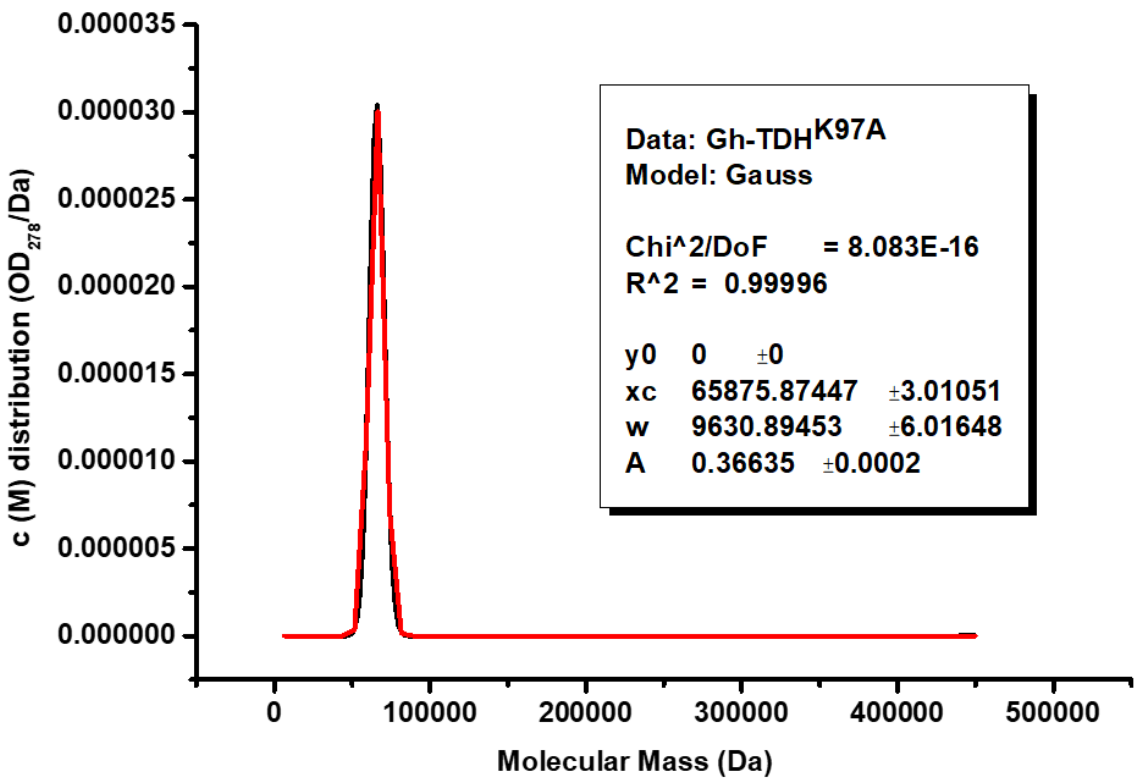

(H) Gh-TDH^{N108A}

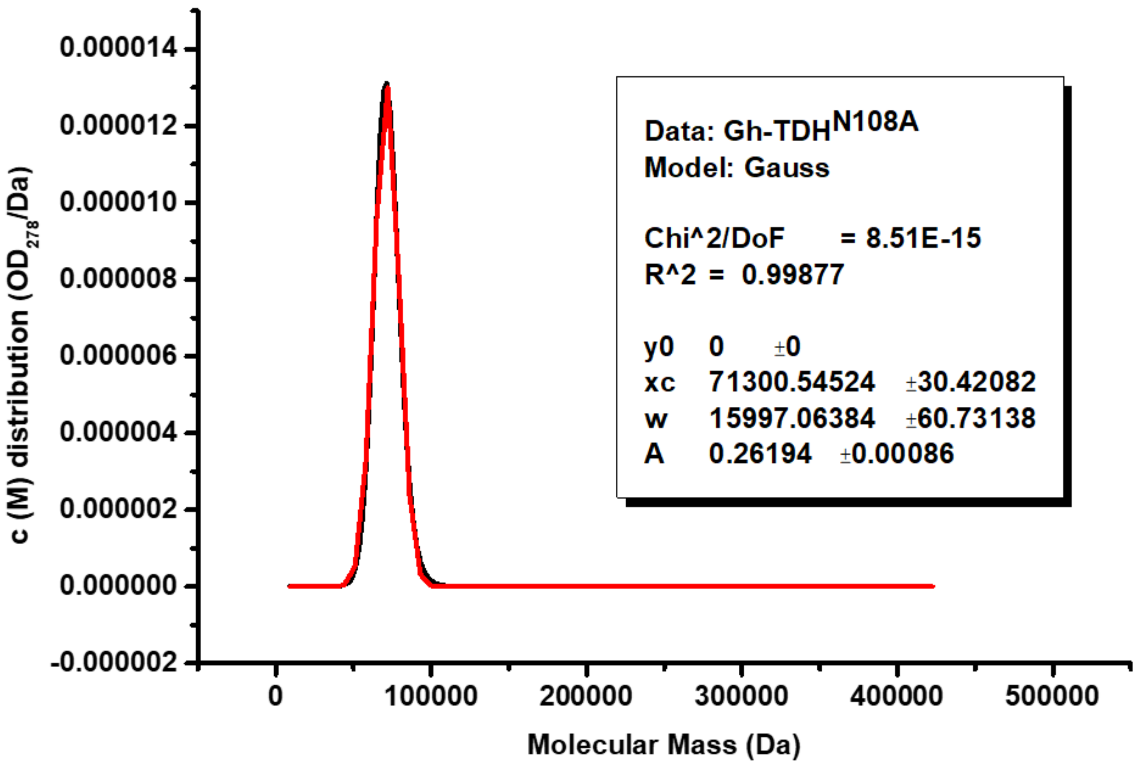

Table S1

MAD data[a]	Gh-TDH-I oligomer			Gh-TDH-II oligomer		
	peak	inflection	remote	peak	inflection	remote
space group		$P2_12_12$			$P2_12_12$	
cell dimension (Å)		105.3, 112.6, 60.8			105.3, 112.7, 60.9	
		α, β, γ = 90º			α, β, γ = 90º	
wavelength	0.9789	0.9791	0.9639	0.9790	0.9792	0.9638
resolution (Å)	30-1.79	50-1.79	30-1.70	30-2.30	30-2.30	30-2.40
	(1.73-1.79)	(1.73-1.79)	(1.70-1.76)	(2.38-2.30)	(2.38-2.30)	(2.38-2.40)
total reflections	700,387	345,485	359,546	263,840	132,647	133,719
unique reflection	71,759	70,365	73,925	32,500	32,652	32,462
R_{merge}	0.077(0.597)	0.073(0.643)	0.064(0.570)	0.083(0.264)	0.080(0.226)	0.068(0.222)
redundancy	9.8 (8.7)[b]	4.9 (4.3)	4.9 (4.1)	8.1 (7.2)	4.1 (3.7)	4.1 (3.8)
I/σ(I)	5.1 (4.8)	4.2 (4.1)	4.3 (4.5)	21.8 (6.7)	14.2 (5.9)	17.0 (6.3)
data completeness (%)	94.4 (93.6)	92.4 (82.5)	92.2 (79.3)	99.6 (97.9)	99.5 (97.4)	99.5 (98.3)
refinement statics						
resolution (Å)		23.85 – 1.70			26.77 – 2.30	
No. reflections		71,897			32,609	
R_{work}/R_{free} (%)		20.11 / 23.36			19.34 / 24.07	
protein atoms		4,817			4,854	
waters		240			184	
B_{factor} (Å^2)						
protein		26.935			31.334	
water		29.428			32.134	
R.M.S. deviation (Å)						
bond lengths (Å)		0.005			0.007	
bond angles (º)		0.847			1.008	
Ramanchandran favored (%)		96.6%			97.25%	
MolProbity score[e] (100th percentile)		0.87			0.95	
All-atom clash score[f] (100th percentile)		0.43			1.06	

a. The multiple anomalous dispersion (MAD) X-ray diffraction data were named based on the Gh-TDH tetrameric structure determined in the crystallographic asymmetric unit.

b. Values in parentheses are parameters for high-resolution shell.

c. $R_{merge} = \sum_{hkl}(\sum_i(|I_{hkl,i}-\langle I_{hkl}\rangle|))/\sum_{hkl,i}\langle I_{hkl}\rangle$, where $I_{hkl,i}$ is the intensity of the reflection with Miller indices h, k and l, and $\langle I_{hkl}\rangle$ is the mean intensity of the reflection.

d. $R_{work} = \sum_{hkl}(||Fobs_{hkl}|-|Fcalc_{hkl}||)/|Fobs_{hkl}|$, where $|Fobs_{hkl}|$ and $|Fcalc_{hkl}|$ are the observed and calculated structure factor amplitudes. R_{free} is calculated with statistically selected ~2000 reflections omitted from the refinement process.

e. MolProbity score is defined as the following:

0.42574*log(1+clashscore)+0.32996*log(1+max(0,pctRotOut-1))+0.24979*log(1+max(0,100-pctRamaFavored-2))+0.5

100th percentile is the best among structures of comparable resolution; 0th percentile is the worst.

f. The clashscore is the number of serious overlaps of non-donor–acceptor atoms by more than 0.4 Å per 1000 atoms.

Table S2. Molecular mass determination of Gh–TDH^{WT} and various Gh–TDH^{mut} proteins.

Mw. (kDa)	WT	Y53D	F159D	R46E	Y53D/ F159D	D78A	K97A	S98A	Q104A	N108A	Y126A	E129A
SDS PAGE	22	22	22	22	22	22	22	22	22	22	22	22
Native-PAGE	90~140	72	72	66	66	90~140	70~95	70~95	70~95	70~95	70~95	70~95
AUC	74.54 ± 0.007	33.09 ± 0.07	28.28 ± 0.15	18.86 ± 0.007	21.88 ± 0.05	72.18 ± 0.02	65.88 ± 0.003	N.D.	N.D.	71.30 ± 0.03	N.D.	N.D.

N. D. = not determined
